# Prevalence and smokers' profile: comparisons between the psychiatric population and the general population*

**DOI:** 10.1590/1518-8345.2976.3149

**Published:** 2019-04-29

**Authors:** Renata Marques de Oliveira, Jair Lício Ferreira Santos, Antonia Regina Ferreira Furegato

**Affiliations:** 1 Faculdade de Medicina de Marília, Hospital das Clínicas de Marília, Marília, SP, Brasil; 2 Universidade de São Paulo, Faculdade de Medicina de Ribeirão Preto, Ribeirão Preto, SP, Brasil; 3 Universidade de São Paulo, Escola de Enfermagem de Ribeirão Preto, Centro Colaborador da OPAS/OMS para o Desenvolvimento da Pesquisa em Enfermagem, Ribeirão Preto, SP, Brasil

**Keywords:** Smoking, Prevalence, Mental Health, Psychiatry, Epidemiology, Psychiatric Nursing, Tabagismo, Prevalência, Saúde Mental, Psiquiatria, Epidemiologia, Enfermagem Psiquiátrica, Tabaquismo, Prevalencia, Salud Mental, Psiquiatría, Epidemiología, Enfermería Psiquiátrica

## Abstract

**Objectives:**

to identify the prevalence of smokers between the psychiatric population and the general population; to compare the personal, socio-demographic and clinical profile of smokers and non-smokers in the psychiatric population and the general population; to compare the reasons for smoking of these two population groups.

**Method:**

this is a cross-sectional descriptive-analytical epidemiological study with 378 patients from three services: Ambulatory Mental Health, Psychiatric Hospital, and Basic Health Unit. Interviews were conducted with three questionnaires. The Chi-square and Kruskal-Wallis tests were applied.

**Results:**

in the total of the 378 participants, 67% were women and 69% were over 40 years old. There was a higher prevalence of smokers among men, young people, illiterates, singles and with more than one government benefit. Smokers prevailed among schizophrenics, chronic patients, who used ≥ 3 psychotropic drugs and had a history of ≥ 4 psychiatric hospitalizations and/or suicide attempts. The main reason for smoking was the improvement of negative feelings.

**Conclusion:**

the prevalence of smokers is higher in the psychiatric population (especially among severely ill patients) and among men, young people, unmarried and with socioeconomic losses. The main reason for smoking is tension/relaxation relief. This study provides nurses and other professionals with knowledge capable of subsidizing the planning of smoking interventions in the Brazilian population.

## Abstract (pt)

**Objetivos:**

identificar prevalência de fumantes entre a população psiquiátrica e a população geral; comparar o perfil pessoal, sociodemográfico e clínico dos fumantes e não fumantes da população psiquiátrica e da população geral; verificar as razões para fumar desses dois grupos populacionais.

**Método:**

estudo epidemiológico descritivo-analítico, de corte transversal, com 378 pacientes de três serviços: Ambulatório Saúde Mental, Hospital Psiquiátrico e Unidade Básica Saúde. Realizaram-se entrevistas com três questionários. Aplicaram-se testes qui-quadrado e *Kruskal-Wallis*.

**Resultados:**

dos 378 participantes, 67% eram mulheres e 69% tinham mais de 40 anos. Identificada maior prevalência de fumantes entre homens, jovens, analfabetos, solteiros e recebedores de mais de um benefício do governo. Os fumantes prevaleceram entre os esquizofrênicos, pacientes crônicos, que utilizavam ≥ 3 psicofármacos e com histórico de ≥ 4 internações psiquiátricas e/ou tentativas suicídio. A principal razão alegada para fumar foi a melhora dos afetos negativos.

**Conclusão:**

a prevalência de fumantes é maior na população psiquiátrica (especialmente entre os pacientes graves) e entre os homens, jovens, solteiros e com prejuízos socioeconômicos. A principal razão para fumar é o alívio da tensão/relaxamento. O presente estudo fornece, aos enfermeiros e demais profissionais, conhecimento capaz de subsidiar o planejamento de intervenções do tabagismo na população brasileira.

## Abstract (es)

**Objetivos:**

identificar prevalencia de fumadores entre la población psiquiátrica y la población general; comparar el perfil personal, sociodemográfico y clínico de los fumadores y no fumadores de la población psiquiátrica y de la población general; verificar las razones para fumar de esos dos grupos poblacionales.

**Método:**

estudio epidemiológico descriptivo-analítico, de corte transversal, con 378 pacientes de tres servicios: Ambulatorio Salud Mental, Hospital Psiquiátrico y Unidad Básica de Salud. Se realizaron entrevistas con tres cuestionarios. Se aplicaron pruebas chi-cuadrado y *Kruskal-Wallis*.

**Resultados:**

de los 378 participantes, 67% eran mujeres y 69% tenían más de 40 años. Identificada mayor prevalencia de fumadores entre hombres, jóvenes, analfabetos, solteros y recibidores de más de un beneficio del gobierno. Los fumadores prevalecieron entre los esquizofrénicos, pacientes crónicos, que utilizaban ≥ 3 psicofármacos y con histórico de ≥ 4 internaciones psiquiátricas y/o tentativas de suicidio. La principal razón alegada para fumar fue la mejoría de los afectos negativos.

**Conclusión:**

la prevalencia de fumadores es mayor en la población psiquiátrica (especialmente entre los pacientes graves) y entre los hombres, jóvenes, solteros y con prejuicios socioeconómicos. La principal razón para fumar es el alivio de la tensión/relajamiento. El presente estudio provee, a los enfermeros y demás profesionales, conocimiento capaz de subsidiar la planificación de intervenciones del tabaquismo en la población brasilera.

## Introduction

Currently, the world prevalence of smokers is 20.7%, while in 2007 it was 23.5%. This result shows an overall trend; however, it is observed that the reduction was more significant in countries with high per capita income^(^ ^1^ ^)^.

More localized studies show a divergence in the prevalence of smokers among different population groups, especially those considered vulnerable - poor, homosexual, with mental disorders, and users of alcohol and illicit substances^(^ ^2^ ^–^ ^4^ ^)^.

Smoking causes about seven million deaths a year, meaning one in 10 deaths by tobacco use. Despite the high mortality rate, 30 million lives may have been saved in the past ten years as a result of the World Health Organization and governments' efforts to control this epidemic^(^ ^1^ ^)^.

For more than a decade, the World Health Organization has proposed actions to control smoking, which include monitoring tobacco use, raising awareness about the harm for the person and passive smokers, encouraging advertisements to be banned tobacco use, tobacco cessation aid, and tobacco tax relief. About two-thirds of the world's population is protected by these actions, as 121 countries adopt at least one of them^(^ ^1^ ^)^.

Although there has been big progress made in recent decades, the World Health Organization recognizes tobacco smoke as a lethal practice, advocating the urgent strengthening of control actions^(^ ^1^ ^)^.

Tobacco smoking among people with mental disorders has always been very frequent and encouraged even by health professionals. Currently, it is seen as a public health problem, since the prevalence of smokers is two or three times higher, compared to the general population. This fact leads to physical losses (high index of early mortality due to clinical comorbidities), mental losses (aggravated by psychiatric symptoms), social losses (social isolation) and financial losses (elimination of essential expenses to buy cigarettes)^(^ ^3^ ^,^ ^5^ ^–^ ^7^ ^)^.

Bringing this discussion to the national level, the last Brazilian survey revealed a prevalence of smokers in the general population of 14.7%, while in 1989, it was 32.4%. Also, over a five-year period (2008 to 2013), the attempts to quit smoking increased from 41.3% to 47.2%, according to a survey of 39,425 Brazilians nationwide^(^ ^8^ ^–^ ^9^ ^)^.

Brazil's commitment to tobacco control is indisputable, as it was one of the first countries to sign the “Framework Convention on Tobacco Control”^(^ ^10^ ^)^. However, control actions are not only modifying the prevalence of smokers but also their distribution.

This is in line with the World Health Organization, which argues that understanding the profile and trends of tobacco smoke contributes to the strengthening of tobacco control policies^(^ ^1^ ^)^.

From this perspective, it is necessary not only to question how many smokers there are, but who current smokers are and their reasons for smoking.

This study aimed to 1) Identify the prevalence of smokers between the psychiatric population and the general population; 2) Compare the personal, socio-demographic and clinical profile of smokers and non-smokers of the psychiatric population and the general population; 3) Verify the reasons to smoke of these two population groups.

## Method

This is a cross-sectional, descriptive-analytical epidemiological study conducted with 378 patients, concomitantly, in three health services in a city of São Paulo: Mental Health Ambulatory, Psychiatric Hospital, and Basic Health Unit.

The Ambulatory of Mental Health and the Psychiatric Hospital were chosen so it was possible to contemplate the population of psychiatric patients, both inpatients and outpatients. The Basic Health Unit was defined as a study place to contemplate the general population.

A simple random probabilistic sample was calculated, estimating that the prevalence of smokers in the mental health ambulatory would be around 40% and 60% in the psychiatric hospital. The prevalence estimated was based on the experience of researchers in mental health services, as well as on the scientific literature^(^ ^11^ ^–^ ^12^ ^)^. With a significance level (α) of 5% and beta (β) of 10%, the sample calculation indicated the need for 126 participants for each study place. Individuals were included in the survey according to the order of arrival at the service or date of hospitalization, during the collection period.

The individual invited to participate should reside in the municipality and be at least 15 years old. Those who had difficulties or were unable to communicate due to vocal or hearing impairment, those who had a diagnosis of mental retardation and who declared problematic use of alcohol or illicit substances without psychiatric comorbidities were excluded.

The same inclusion and exclusion criteria were considered for the population of the Ambulatory of Mental Health, the Psychiatric Hospital, and the Basic Health Unit. Therefore, it was decided not to exclude people with mental disorders from the Basic Health Unit to maintain comparability with studies conducted by the World Health Organization and by other authors, who do not use the psychiatric diagnosis as an exclusion criterion to investigate smoking in the general population^(^ ^1^ ^,^ ^8^ ^–^ ^9^ ^)^.

**Figure 1:**
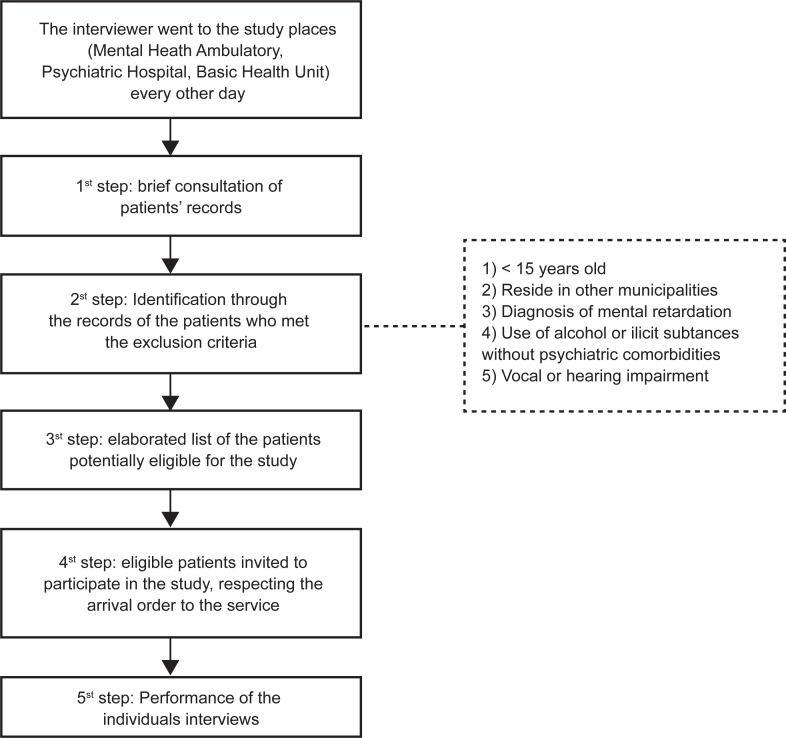
Steps of the definition of patients included in the study

In Figure 1, the process of study participants definition is illustrated:

The project was registered in the Brazil/CONEP Platform (CAAE 21101113.3.0000.5393) and approved by the Research Ethics Committee of the Ribeirão Preto Nursing College (308/2013). Participants signed two copies of the Informed Consent Form (ICF), and a copy was filed with the researchers. Participants between 15 and 18 years old (n=3) signed a consent agreement and their guardians an ICF authorized the participation of the minor.

The data were obtained by a single researcher, from individual interviews with the 378 participants, in a reserved room. The interviews had an average duration of 18 minutes (10 to 47 minutes). Three instruments were used: 1) Identification questionnaire for patients attending mental health and basic care services, 2) Psychiatric Evaluation Brief Scale, and 3) Modified Smoking Reason Scale. The instruments were scanned in the TabacoQuest application and respondents' answers were recorded on a mobile device^(^ ^13^ ^)^.

The patient identification questionnaire was specially designed for the project of this article, and 15 variables were selected. The outcome variable is “current tobacco smoking” with the dichotomous smoking and non-smoking categories. The other variables were selected to compare the profile of smokers and non-smokers: sex (female, male); age group (15 to 29 years old, 30 to 39 years old, 40 to 49 years old, 50 to 59 years old, ≥ 60 years old); education level (illiterate, elementary school, high school, higher education); marital status (single, married, separated/divorced, widow); home arrangement (living alone, without roommate/family, without roommate/with other people, with roommate only, with roommate and family); occupation (retired, housewife, worker, without occupation); abandonment of the employment relationship after the diagnosis of the mental disorder (yes, no, it does not apply); government benefits (none, one, two or more); psychiatric diagnosis (schizophrenia/schizoaffective disorder); time of diagnosis (<1 year, 1 to 12 years,> 12 years); psychotropic drugs in use (none, one, two, three, four, between five and seven); current use of antipsychotics (first generation, second generation, first and second generation, it does not apply); psychiatric hospitalizations (none, one, two, three, four or more); suicide attempts (none, one, two, three, four or more).

The “Brief Scale of Psychiatric Evaluation” was used to compare the psychiatric symptomatology of smokers and non-smokers, evaluating the presence and severity of 18 symptoms in the last three days - somatic concerns; psychic anxiety; emotional withdrawal; conceptual disorganization (incoherence); guilt; somatic anxiety; specific motor disturbances; exaggerated self-esteem; depressed mood; hostility; suspiciousness; hallucinations; motor retardation; uncooperativeness; unusual thought content; blunted affect; motor hyperactivity and disorientation. The evaluation of the severity of each symptom (0 = absent, 1 = very mild or with dubious presence, 2 = present in mild degree, 3 = present in moderate degree, 4 = present in severe or extreme degree). The within-class reliability was 0.93^(^ ^14^ ^)^.

The “Reasons for Modified Smoking Scale” was developed to evaluate the reasons people smoke tobacco^(^ ^15^ ^)^. It is composed of 21 affirmations and evaluates seven factors: 1) dependence; 2) pleasure of smoking; 3) reduction of tension/relaxation; 4) social smoking; 5) stimulation; 6) habit/automatism and 7) handling. Participants indicate how much each statement applies to their daily life: (1) never; (2) rarely; (3) sometimes; (4) often and (5) always.

Statistical analysis was performed in Stata/SE (version 12.1). Absolute and relative frequency (%) was calculated using the chi-square test, at the significance level (α) of 5%. The chi-square test was used to identify statistical evidence of an association between the variable “current tobacco smoke” and the other variables tested two to two.

Although the “Brief Scale of Psychiatric Evaluation” provides five possible classifications for symptoms - 1) absent, 2) very mild or with dubious presence, 3) present in mild degree, 4) present in a moderate degree and 5) present in severe or extreme degree^(^ ^14^ ^)^, due to the sample size, for the statistical analysis of this study, three categories were chosen for each symptom: 1) absent; 2) very light, dubious or light presence; 3) moderate to severe extreme.

The Cramér coefficient V was applied to estimate the strength of the association in cases where the chi-square test indicated p<0.05. The classification of the association was weak, moderate and strong.

Finally, the answers of the 134 smokers on the “Reasons for Modified Smoking” scale were analyzed. Each of the seven domains evaluated by the scale (reduction of tension/relaxation, dependence, the pleasure of smoking, handling, stimulation, social smoking and habit/automatism) were considered as outcome variables. The Kruskal-Wallis test was applied to analyze domains (reasons for smoking) according to the study sites (Mental Health Ambulatory, Psychiatric Hospital, and Basic Health Unit).

The results were discussed based on the scientific literature on this topic.

## Results

Of the total of 378 participants, 67% were women, 69% were over 40 years old and 56% had studied through elementary school. In the Basic Health Unit, 29% had a psychiatric diagnosis, recorded in their medical records. In the Mental Health Ambulatory and in the Psychiatric Hospital, this percentage was 100%.

The prevalence of smokers was different in the three places investigated (ambulatory=27%, hospital =60%, basic health unit=19%).

Table 1 shows the personal and socio-demographic characteristics of smokers (n=134) and non-smokers (n=244).

**Table 1:** Absolute and relative frequency (%) of the identification and socio-demographic profile of the participants, according to tobacco smoke (n=378). Marília, SP, Brazil, 2016

Variables		Smoker	Non-smoker	Total	X^2^ p-value (strength association)
		n (%)	n (%)	N	
Gender					X^2^= 15.9395 p < 0.001* (weak)
	Female	73 (28.6)	182 (71.4)	255	
	Male	61 (49.6)	62 (50.4)	123	
Age group (years old)					X^2^= 6.6642 p= 0.155
	15 to 29	22 (45.8)	26 (54.2)	48	
	30 to 39	25 (36.8)	43 (63.2)	68	
	40 to 49	29 (40.3)	43 (59.7)	72	
	50 to 59	35 (34.6)	66 (65.4)	101	
	≥ 60	23 (25.8)	66 (74.2)	89	
Education level					X^2^= 6.4728 p= 0.091
	Illiterate	7 (35)	13 (65)	20	
	Elementary school	86 (40.6)	126 (59.4)	212	
	High school	32 (29.9)	75 (70.1)	107	
	Higher education	9 (23.1)	30 (76.9)	39	
Marital status					X^2^= 22.0985 p < 0.001* (weak)
	Single	74 (48)	80 (51.9)	154	
	Married	29 (21.8)	104 (78.2)	133	
	Separated/divorced	20 (37.0)	34 (63)	54	
	Widow	11 (29.7)	26 (70.3)	37	
Home arrangement					X^2^= 13.0584 p= 0.011* (weak)
	Living alone	19 (38.8)	30 (61.2)	49	
	Without companion, with relatives	68 (42.8)	91 (57.2)	159	
	Without a partner, with other people	5 (62.5)	3 (37.5)	8	
	With a partner only	13 (24.1)	41 (75.9)	54	
	With partner and family	29 (26.8)	79 (73.2)	108	
Occupation					X^2^= 10.2195 p= 0.017* (weak)
	Retired	40 (40.4)	59 (59.6)	99	
	Housewife	26 (29.2)	63 (70.8)	89	
	Worker	30 (27.5)	79 (72.5)	109	
	No occupation	38 (46.9)	43 (53.1)	81	
Abandonment of the employment relationship after diagnosis of mental disorder					X^2^= 25.6849 p < 0.001* (weak)
	Yes	74 (49.3)	76 (50.7)	150	
	No	44 (31.9)	94 (68.1)	138	
	It does not apply	16 (17.8)	74 (82.2)	90	
Benefits of government					X^2^= 0.2250 p= 0.894
	None	67 (35.6)	121 (64.4)	188	
	One	59 (34.7)	111 (65.3)	170	
	Two or more	8 (40)	12 (60)	20	
	Total	134 (35.4)	244 (64.6)	378	

* evidence of statistical association (p< 0.05)

Data showed that while approximately half of the men smoked tobacco, most of the women were non-smokers. Non-smokers prevailed at all ages, but their highest frequency was noted in older people (≥ 60 years old). The prevalence of smokers was higher in young people (15 to 29 years old) and decreased as the aging process.

The highest prevalence of smokers was identified in the illiterate and in those who studied until elementary school. The non-smokers prevalence was identified in those with higher education.

While non-smokers were mostly married, separated/divorced and widowed, nearly half singles were smokers. Consistent with marital status, the highest prevalence of smokers were identified in those who lived without a partner.

There was a higher prevalence of smokers in those participants without occupation and in the retirees. The prevalence of non-smokers was higher among workers and housewives.

The prevalence of smokers was higher in those who stated that they did not have a current occupation, as it was higher in those who received more than one government benefit. Half of the smokers reported having abandoned some employment relationship when diagnosed with mental disorder.

Regarding the clinical profile, a higher prevalence of smokers was observed in people diagnosed with schizophrenia/schizoaffective disorder, followed by those with personality disorders. Non-smokers predominated in those without a psychiatric diagnosis, with mood disorders and with anxious disorders (Table 2).

**Table 2:** Absolute and relative frequency (%) of the clinical profile of the participants of this study, according to tobacco smoke (n=378). Marília, SP, Brazil, 2016

Variables		Smokers	Non-smokers	Total	X^2^ p-value (strength association)
		n (%)	n (%)	N	
Psychiatric diagnosis					X^2^= 37.4027 p < 0.001* (moderate)
	Schizophrenia/schizoaffective	62 (56.4)	48 (43.6)	110	
	Mood Disorders	20 (27.8)	52 (72.2)	72	
	Personality Disorders	16 (43.2)	21 (56.8)	37	
	Anxiety disorders	20 (29)	49 (71)	69	
	No diagnosis	16 (17.8)	74 (82.2)	90	
Diagnostic time (years)					X^2^= 30.9847 p < 0.001* (moderate)
	< 1	5 (13.2)	33 (86.8)	38	
	1 a 12	57 (46)	67 (54)	124	
	> 12	56 (44.4)	70 (55.6)	126	
Psychotropic drugs in use					X^2^= 22.8938 p < 0.001* (moderate)
	None	16 (18.4)	71 (81.6)	87	
	One	13 (25)	39 (75)	52	
	Two	35 (38.9)	55 (61.1)	90	
	Three	40 (47.6)	44 (52.4)	84	
	Four	22 (47.8)	24 (52.2)	46	
	Between five and seven	8 (42.1)	11 (57.9)	19	
Current use of antipsychotics					X^2^= 43.5395 p < 0.001* (moderate)
	First generation	56 (61.5)	35 (38.5)	91	
	Second generation	19 (33.3)	38 (66.7)	57	
	First and second generation	19 (42.2)	26 (57.8)	45	
	Not applicable	40 (21.6)	145 (78.4)	185	
Psychiatric Hospitalizations					X^2^= 51.7600 p < 0.001* (moderate)
	None	39 (21.8)	140 (78.2)	179	
	One	13 (25)	39 (75)	52	
	Two	12 (38.7)	19 (61.3)	31	
	Three	6 (40)	9 (60)	15	
	Four or more	64 (63.4)	37 (36.6)	101	
Attempted suicide					X^2^= 13.2887 p= 0.010* (moderate)
	None	80 (30.3)	184 (69.7)	264	
	One	13 (37.1)	22 (62.9)	35	
	Two	11 (47.8)	12 (52.2)	23	
	Three	12 (48)	13 (52)	25	
	Four or more	18 (58.1)	13 (41.9)	31	
	Total	134 (35.4)	244 (64.6)	378	

* evidence of statistical association (p< 0,05)

As observed in Table 2, the vast majority of those who had been diagnosed for less than one year were non-smokers, among the 288 patients with mental disorders. Smokers prevailed among those with longer diagnosis time. However, there was no significant difference between the participants diagnosed between 12 years old and younger and those diagnosed for more than 12 years.

The highest prevalence of smokers was among those who used three or more psychotropic drugs and 1^st^ generation antipsychotics.

There was a difference in the history of psychiatric hospitalizations, according to the use of tobacco. Most who had never been hospitalized were non-smokers, while the majority of those who had had four or more hospitalizations were smokers. As the number of hospitalizations increased, the prevalence of smokers increased and non-smokers decreased.

While most participants who had never tried suicide did not smoke tobacco, most who had tried four or more times were smokers.

When assessing the presence and severity of psychiatric symptoms, during the three days before the interview, smokers had the most severe symptoms. Three-quarters of the respondents classified in the total score of the “Brief Psychiatric Assessment Scale” as “major syndrome” smoked tobacco.

In Table 3, the psychiatric symptomatology related to thinking, sensory perception and behavior is compared between smokers and non-smokers.

**Table 3:** Absolute and relative frequency (%) of the severity of psychiatric symptoms related to thinking, sensing and behavior, presented during the three days before the interview, according to tobacco smoke (n=378). Marília, SP, Brazil, 2016

Variables		Smokers n (%)	Non-smokers n (%)	Total N	X^2^ p-value (strength association)
Total BPRS score*					X^2^=15.8631 p < 0.001† (moderate)
	Absent syndrome	93 (30.7)	210 (69.3)	303	
	Minor syndrome	38 (53.5)	33 (46.5)	71	
	Major syndrome	3 (75)	1 (25)	4	
Somatic Concerns					X^2^= 1.5816, p= 0.209
	Absent	119 (34.5)	226 (65.5)	345	
	Very light, dubious or slight presence	14 (43.8)	18 (54.5)	32	
	Moderate to Severe/Extreme	1 (100)		1	
Conceptual disorganization					X^2^= 0.0150, p= 0.902
	Absent	131 (35.4)	239 (64.6)	370	
	Very light, dubious or slight presence	3 (37.5)	5 (62.5)	8	
Suspiciousness					X^2^= 6.1241 p= 0.047† (moderate)
	Absent	95 (33.3)	190 (66.7)	285	
	Very light, dubious or slight presence	32 (38.5)	51 (61.5)	83	
	Moderate to Severe/Extreme	7 (70)	3 (30)	10	
Unusual thought content					X^2^= 7.4941 p= 0.024† (moderate)
	Absent	107 (32.8)	219 (67.2)	326	
	Very light, dubious or slight presence	25 (53.2)	22 (46.8)	47	
	Moderate to Severe/Extreme	2 (40)	3 (60)	5	
Hallucinations					X^2^=17.1682 p< 0.001† (moderate)
	Absent	101 (32.4)	211 (67.6)	312	
	Very light, dubious or slight presence	18 (38.3)	29 (61.7)	47	
	Moderate to Severe/Extreme	15 (79)	4 (21)	19	
Specific motor disturbances					X^2^= 1.8307 p= 0.400
	Absent	132 (35.3)	242 (64.7)	374	
	Very light, dubious or slight presence		1 (100)	1	
	Moderate to Severe/Extreme	2 (66.7)	1 (33.3)	3	

* BPRS: Brief Psychiatric Evaluation Scale^(14)^;

† evidence of statistical association (p<0.05)

In Table 4, the psychiatric symptoms of the “Brief Scale of Psychiatric Assessment”, related to orientation, anxiety and mood/affection are detailed.

**Table 4:** Absolute and relative frequency (%) of the severity of psychiatric symptoms related to orientation, anxiety, mood/affection, presented during the three days prior to the interview, according to the use of tobacco (n = 378). Marília, SP, Brazil, 2016

Variables*		Smokers	Non-smokers	Total	p-value
		n (%)	n (%)	N	
Psychic anxiety					X^2^=8.6005, p= 0.014† (moderate)
	Absent	51 (29.1)	124 (70.9)	175	
	Very light, dubious or slight presence	62 (38)	101 (62)	163	
	Moderate to Severe/Extreme	21 (52.5)	19 (47.5)	40	
Somatic anxiety					X^2^=10.5208 p= 0.005† (moderate)
	Absent	86 (32.6)	178 (67.4)	264	
	Very light, dubious or slight presence	37 (37.4)	62 (62.6)	99	
	Moderate to Severe/Extreme	11 (73.3)	4 (26.7)	15	
Blunted affect					X^2^= 2.9418 p= 0.230
	Absent	132 (36.3)	232 (63.7)	364	
	Very light, dubious or slight presence	2 (15.4)	11 (84.6)	13	
	Moderate to Severe/Extreme		1 (100)	1	
Guilt					X^2^=20.1759 p < 0.001† (moderate)
	Absent	86 (29.4)	206 (70.6)	292	
	Very light, dubious or slight presence	48 (55.8)	38 (44.2)	86	
Exaggerated self-esteem					X^2^=4.3541 p= 0.001† (moderate)
	Absent	116 (32.9)	236 (67.1)	352	
	Very light, dubious or slight presence	17 (68)	8 (32)	25	
	Moderate to Severe/Extreme	1 (100)		1	
Depressed mood					X^2^= 7.2664 p= 0.026 (moderate)
	Absent	57 (29.1)	139 (70.9)	196	
	Very light, dubious or slight presence	69 (42.6)	93 (57.4)	162	
	Moderate to Severe/Extreme	8 (40)	12 (60)	20	
Emotional withdrawal					X^2^= 8.3814 p= 0.015† (moderate)
	Absent	89 (31.4)	194 (68.6)	283	
	Very light, dubious or slight presence	43 (46.7)	49 (53.3)	92	
	Moderate to Severe/Extreme	2 (66.7)	1 (33.3)	3	
Disorientation					X^2^=20.0854 p < 0.001† (moderate)
	Absent	93 (30.3)	214 (69.7)	307	
	Very light, dubious or slight presence	30 (54.5)	25 (45.5)	55	
	Moderate to Severe/Extreme	11 (68.8)	5 (31.2)	16	
Total		134 (35.4)	244 (64.5)	378	

* Variables: symptoms evaluated from the “Brief Psychiatric Assessment Scale”^(14)^;

† Evidence of statistical association (p< 0.05)

Few participants had blunted affect (n=14), conceptual disorganization (incoherence) (n=8), specific motor disturbances (n=4), motor retardation (n=5), uncooperativeness (n=6) and motor hyperactivity (n=8). This result was expected, considering that people with these changes would hardly be able to participate in the interviews.

Among the domains evaluated by the “Reasons for Modified Smoking Scale” in the 134 smokers, smoking predominated as an aid to deal with negative feelings (reduction of tension/relaxation), having even exceeded what they evaluated tobacco dependence: 1) reduction of tension/relaxation (mean = 3.7, standard deviation = 1.2); 2) dependence (mean=3.6, standard deviation=1.3); 3) smoking pleasure (mean=3.5, standard deviation=1.1); 4) handling (mean=3.1, standard deviation=1.4); 5) stimulation (mean=3.0, standard deviation=1.4); 6) social smoking (mean=2.8, standard deviation=1.4) and 7) habit/automatism (mean=2.4, standard deviation=1.2).

Comparing the “Reasons for Modified Smoking Scale” domains in smokers from the Mental Health Clinic, the Psychiatric Hospital and the Basic Health Unit, the Kruskal-Wallis test indicated a difference only when comparing the “handling” domain (p=0.043), and the mean score for smokers in the psychiatric hospital was higher (3.3) than smokers in the outpatient clinic (3.1) and the primary care unit (2.5).

## Discussion

This study identified that smokers are predominantly male, young and single, and those with socioeconomic losses (illiterate or with few years of school, people with no employment relationship and receiving social benefits from the government).

This study is in line with the scientific literature regarding people with socioeconomic vulnerability, more likely to use tobacco^(^ ^16^ ^–^ ^19^ ^)^. However, it is a vicious cycle in which social disadvantages make people more vulnerable to smoking, and becoming a smoker contributes to these disadvantages (smokers stop buying essential items such as food and medicine to buy cigarettes)^(^ ^18^ ^)^.

An American longitudinal study with 131 smokers and 120 non-smokers, looking for work, helps to understand this situation. Almost half of the smokers (45.8%) reported having been discriminated against in previous jobs by smoking tobacco and 8.4% admitted to having been dismissed for that reason. Although 29% acknowledged that being a smoker hindered to get a new job, tobacco purchases were listed as the highest financial priority, even exceeding food expenses^(^ ^19^ ^)^.

After a follow up of 12 months, those who did not smoke were more successful (55.6%) in re-entering the labor market than smokers (26.6%). If the 131 smokers stopped smoking, the percentage of reemployment would increase by 30%, regardless of unemployment time, age, school years, race/ethnicity and health conditions^(^ ^19^ ^)^.

Socioeconomic vulnerability helps to understand, in part, the lower prevalence of smokers in the mental health ambulatory compared to the psychiatric hospital. Because psychiatric treatment is too expensive (as an example, each psychiatric visit is charged without return visits as in other specialties), it is common to find people with good economic conditions in mental health services.

Regarding the clinical profile, this study revealed a higher prevalence of smokers among the more severe psychiatric patients (diagnosis of schizophrenia or schizoaffective disorder), with intense symptoms, with a longer diagnosis, using three or more psychotropic drugs, especially antipsychotics of first generation, with a history of four or more psychiatric hospitalizations, as well as four or more suicide attempts.

The clinical profile of smokers was similar to the predominant characteristics of the participants in the psychiatric hospital, coincidentally, where there was the highest prevalence of smokers, in relation to the others.

As found in this study, the higher prevalence of smokers among schizophrenics, compared to those diagnosed with other mental disorders, is widely recognized in the scientific literature^(^ ^3^ ^,^ ^5^ ^–^ ^6^ ^,^ ^20^ ^)^.

The theory of self-medication exposes that tobacco would improve the cognitive symptoms of schizophrenia by increasing the release of dopamine and glutamate in the prefrontal cortex and by regulating the auditory sensory process so the schizophrenic can filter out those irrelevant stimuli from the environment that harm their cognitive functions (attention, concentration, memory).

Negative symptoms (anecdotal, affective blunting, psychomotor retardation, loss of initiative) would be ameliorated by the ability of tobacco to act on deficits in the brain reward system, commonly presented by schizophrenics, justifying the greatest cleavage among them^(^ ^6^ ^,^ ^21^ ^–^ ^22^ ^)^.

The data in this study lead to think of the verisimilitude of this theory, since almost two-thirds of those using only the first-generation antipsychotics were smokers, while the majority of those using only second-generation antipsychotics were non-smokers.

This finding is consistent with the scientific literature showing that individuals on first-generation antipsychotics are more likely to use tobacco than those on second-generation antipsychotics^(^ ^23^ ^–^ ^25^ ^)^.

Therefore, first-generation antipsychotics act only on positive symptoms (delusions, hallucinations, among others). The schizophrenic, using this type of psychoactive drug, would find in tobacco a way of temporarily reversing cognitive symptoms by inducing an increase in dopamine and glutamate in the prefrontal cortex^(^ ^6^ ^,^ ^26^ ^–^ ^28^ ^)^. According to the self-medication theory, the lower prevalence of smokers in people using only second-generation antipsychotics would be justified because this class of psychotropic drugs acts on both the positive and negative symptoms of schizophrenia. Therefore, as a form of self-medication of cognitive symptoms, tobacco smoke is not an argument used by people with mental disorders using second-generation antipsychotics.

Despite the hypothetical improvement of the negative and cognitive symptoms with the use of tobacco, there is evidence that tobacco aggravates the positive symptoms of schizophrenia by increasing the dopaminergic activity in the mesolimbic region, which would justify in part the worse prognosis identified between the smokers^(^ ^18^ ^,^ ^22^ ^,^ ^29^ ^)^.

In fact, a significant portion of the people who presented delusions and hallucinations, in the days before the interview, were smokers.

Regardless of whether the theory of self-medication is true, smokers were those who presented more intense psychiatric symptoms (total score in the “Brief Psychiatric Evaluation Scale”). These results were consistent with other studies^(^ ^22^ ^,^ ^30^ ^–^ ^31^ ^)^.

The greater intensity of psychiatric symptoms among smokers is in agreement with some theories that although there may be an improvement of the negative, cognitive and anxiety symptoms at the onset of smoking, chronic use of tobacco can reverse this effect, increasing the symptoms^(^ ^18^ ^,^ ^29^ ^,^ ^32^ ^–^ ^33^ ^)^.

Complementing the theory of self-medication, studies showed that tobacco use interferes with the metabolism of psychoactive drugs, decreasing its concentration in plasma. Therefore, psychiatric patients would use tobacco more intensely as a way to alleviate side effects, especially in the case of first-generation antipsychotics^(^ ^6^ ^,^ ^22^ ^,^ ^34^ ^)^.

In the same line, the Brazilian study in the General Hospital Psychiatric Unit found that 50% of smokers with a diagnosis of schizophrenia justified the maintenance of smoking with the intention of alleviating the side effects of psychotropic drugs^(^ ^35^ ^)^.

Tobacco interference in the metabolism of psychotropic drugs would explain the more intense psychiatric symptoms, the greater the quantity of prescribed psychotropic drugs, the greater the occurrence of psychiatric hospitalizations and suicide attempts.

The relationship between smoking and psychiatric hospitalizations is a recurrent topic in the scientific literature. Regardless of other factors, the Brazilian study conducted in psychiatric hospitals and Psychosocial Care Centers in the five regions of the country found that the prevalence of smokers is 69% higher among patients with a previous history of psychiatric hospitalization compared to non-smokers^(^ ^36^ ^)^. In this study, suicide attempts were highlighted, since the prevalence of smokers increased according to the number of attempts. Non-smokers followed the opposite direction of smokers.

This result is corroborated by other studies^(^ ^37^ ^–^ ^39^ ^)^. The relationship between suicide and smoking was highlighted when cohort studies identified tobacco use and its high dependence as a risk factor for suicidal behavior, even after adjusting for psychiatric variables.

There is evidence of a dose-response effect as the higher the number of cigarettes smoked/day, the greater the risk of suicide. Moreover, there was evidence of a decrease in this risk when stopping smoking^(^ ^37^ ^–^ ^39^ ^)^.

The number of psychiatric hospitalizations, as well as the number of suicide attempts and prescribed psychotropic drugs, are variables that indirectly portray the time of psychiatric diagnosis.

Therefore, independently of the proof of the theory of self-medication and of tobacco interference in the action of psychoactive drugs, the greater number of hospitalizations, suicide attempts and psychotropic drugs expresses the severity and chronicity of mental disorders, since there is no doubt that the prevalence of smokers is more significant among those with more time to diagnosis, as confirmed in other studies^(^ ^4^ ^,^ ^35^ ^,^ ^40^ ^)^.

The main reason as the motivation of smokers who participated in this study to use tobacco was the reduction of tension/relaxation.

The use of tobacco as an attempt to alleviate anxiety is known. A Scottish study of 131 schizophrenics showed that 60% of smokers used tobacco to relax and 31% because they felt anxious or depressed^(^ ^22^ ^)^. Similarly, the Brazilian study with 270 psychiatric patients revealed that 79% of smokers believed in the anxiolytic function of tobacco^(^ ^35^ ^)^.

Despite the above, special care is needed for these results. While it is still possible to identify people who use tobacco to feel less anxious and safer in social interactions, this is a reality that is being modified, as tobacco smoke is moving from a glamorous act to conduct condemned by society^(^ ^41^ ^)^.

With the higher prevalence of smokers in the psychiatric population and the lower tolerance of society to smoking in collective settings, the trend is that those with mental disorders are even more discriminated and excluded from social interaction.

This study provides nurses and other health professionals with elements for a better understanding of smoking since it presents the personal, socio-demographic and clinical profile and the reasons for these people to smoke cigarettes. This can contribute to the planning of future interventions throughout the network of health services, educational programs and direct care for the mentally ill people. Also, this study provides nurses with new knowledge, since the research topic has been little investigated in Brazilian scientific literature.

Limitations: 1) sample restricted to people assisted in the health services of a single municipality in the interior of São Paulo; 2) no multivariate analysis was performed.

## Conclusion

The prevalence of smokers is higher in the psychiatric population, especially the hospitalized population.

When considering the psychiatric population and the general population, the study identified that smokers are predominantly male, young and single, and with socioeconomic losses (illiterate or with few years of school, people with no employment receive social benefits from the government).

Regarding the clinical profile, this study revealed a higher prevalence of smokers among the more severe psychiatric patients (diagnosis of schizophrenia or schizoaffective disorder), with intense symptoms, with a longer diagnosis, using three or more psychotropic drugs, especially antipsychotics of first generation, with a history of four or more psychiatric hospitalizations, as well as four or more suicide attempts.

The main reason alleged to justify maintaining cigarette smoke is to obtain tension relief and relaxation.

It is expected that this study will provide nurses and other health professionals with knowledge capable of subsidizing educational projects, as well as planning smoking interventions in the Brazilian psychiatric population.
